# Microglia Contribution to the Regulation of the Retinal and Choroidal Vasculature in Age-Related Macular Degeneration

**DOI:** 10.3390/cells9051217

**Published:** 2020-05-14

**Authors:** C. Henrique Alves, Rosa Fernandes, Ana Raquel Santiago, António Francisco Ambrósio

**Affiliations:** 1 Retinal Dysfunction and Neuroinflammation Lab, Coimbra Institute for Clinical and Biomedical Research (iCBR), Faculty of Medicine, University of Coimbra, 3000-548 Coimbra, Portugal; chalves@fmed.uc.pt (C.H.A.); rcfernandes@fmed.uc.pt (R.F.); asantiago@fmed.uc.pt (A.R.S.); 2 Center for Innovative Biomedicine and Biotechnology (CIBB), University of Coimbra, 3004-531 Coimbra, Portugal; 3 Association for Innovation and Biomedical Research on Light and Image (AIBILI), 3000-548 Coimbra, Portugal; 4 Clinical Academic Center of Coimbra (CACC), 3004-561 Coimbra, Portugal

**Keywords:** age-related macular degeneration (AMD), retina, choroid, microglia, retinal vasculature, inflammation, neogenesis, angiogenesis

## Abstract

The retina is a highly metabolically active tissue with high-level consumption of nutrients and oxygen. This high metabolic demand requires a properly developed and maintained vascular system. The retina is nourished by two systems: the central retinal artery that supplies the inner retina and the choriocapillaris that supplies the outer retina and retinal pigment epithelium (RPE). Pathological neovascularization, characterized by endothelial cell proliferation and new vessel formation, is a common hallmark in several retinal degenerative diseases, including age-related macular degeneration (AMD). A limited number of studies have suggested that microglia, the resident immune cells of the retina, have an important role not only in the pathology but also in the formation and physiology of the retinal vascular system. Here, we review the current knowledge on microglial interaction with the retinal vascular system under physiological and pathological conditions. To do so, we first highlight the role of microglial cells in the formation and maintenance of the retinal vasculature system. Thereafter, we discuss the molecular signaling mechanisms through which microglial cells contribute to the alterations in retinal and choroidal vasculatures and to the neovascularization in AMD.

## 1. Introduction

The retina is the nervous tissue that sits at the back of the eye and is responsible for converting light into electrical signals and sending these on to the brain for visual recognition. Retinal development is a long and complex process that, in humans, begins during the fourth week of embryogenesis and continues into the first year of life [1]. During retinogenesis, retinal progenitor cells give rise to six major types of neurons, ganglion cells, amacrine cells, horizontal cells, bipolar cells, cone and rod photoreceptor cells and one glial cell type, the Müller glial cells [2]. The six different cell lines are organized into a laminated structure composed of three nuclear layers, the outer nuclear layer comprising the cell soma of the photoreceptors; the inner nuclear layer composed by the nuclei of the horizontal, bipolar and amacrine cells and of the Müller glial cells; and the ganglion cell layer (GCL) consisting of the soma of ganglion and amacrine cells. The three nuclear layers are divided by two plexiform layers composed by the axonal, dendritic processes and synapses of these cells [3].

Microglia, the resident immune cells of the retina, are not derived from retinal progenitors cells but are derived from primitive yolk sac progenitors [4], and therefore are from mesodermal/mesenchymal origin. The precursors of microglia are found in the retina, before vascularization, via the vitreal surface of the retina or by migrating from non-neural ciliary regions in the periphery [5,6]. In the adult retina, microglial cells are distributed in the plexiform layers, ganglion cell layer and nerve fiber layer, where they survey the surrounding environment with their motile processes [7,8].

With approximately 4.5 million cone and 90 million rod photoreceptors, the human retina is the most metabolically expensive tissue in the human body. The retina is fed nutrients and oxygen from a unique dual blood supply that divides the retina into outer and inner layers [9]. The formation of the retinal vasculature is a timely controlled process driven by numerous signaling pathways and cellular interactions. Pathological blood vessels in the eye constitute a threat to normal vision. Angiogenesis, important in both physiological vascular development and pathological neovascularization, occurs as endothelial cells proliferate and form new vessels following guidance cues and angiogenic stimulators and inhibitors [10]. Dysregulated angiogenesis disrupts delivery of oxygen and nutrients, resulting in unbalanced metabolic demand and supply and disturbed neural retinal function. Abnormal ocular angiogenesis is associated with a broad spectrum of eye diseases, including neovascular age-related macular degeneration (AMD) [11,12], diabetic retinopathy [12,13], retinopathy of prematurity [14], retinitis pigmentosa [15], amongst others. Pathological retinal neovascularization is characterized by leaky and tuft-like vessels, which are associated with retinal exudates and hemorrhages, that might lead to retinal detachment, retinal damage or both [16].

The retina has the particularity of being the only tissue in the human body for which deep vasculature can be visualized directly and in a non-invasive way using, for example, optical coherence tomography angiography (OCTA), presenting a unique opportunity to study vascular alterations in eye diseases [17,18].

Several studies demonstrated the importance of microglial cells in retinal development and degeneration. However, recently, some studies suggested that microglia also play an important role in the development of retinal vasculature [8,19,20,21,22]. Here, we will focus on the molecular and cellular mechanisms ruled by microglia and their contribution to the formation and maintenance of the retinal vasculature under physiological and disease conditions.

## 2. Development and Structure of Retinal and Choroidal Vasculature

The development of the vasculatures of the embryonic and fetal human eye is an orchestrated and synchronous process that is dependent on the demand for oxygen. The choroidal vasculature is the first to develop followed by the hyaloid vasculature and then the retinal vasculature [23].

The choroid is a thin, highly vascularized and pigmented tissue positioned under the neural retina that constitutes the posterior part of the uveal tract (the iris, ciliary body and choroid). The inner limit of the choroid is the Bruch’s membrane on which the retinal pigment epithelium (RPE) lies [2]. The vascular layer of the choroid is divided into three layers from internal to external, with increasing luminal diameter: the anterior choriocapillaris with broad and flat lumens arranged in a honeycomb-like lobular pattern especially in the posterior pole, Sattler’s layer of intermediate vessels in the middle and the outermost Haller’s layer with large vessels [24].

The main function of the choroid is to nourish the outer retina, RPE, the foveal avascular zone and the optic nerve [25], maintaining the metabolic demands of the RPE and photoreceptor cells. Being the largest source of blood supply to the posterior segment of the eye, the choroidal vasculature is also responsible for the transport of metabolic waste from the RPE, contributing to the normal retinal function. The choroid also acts as a thermal regulator for the outer retina via heat dissipation [26,27]. The choroidal circulation supplies the inner retina, in species where the retinal vessels are absent (guinea pig) or sparse (rabbit) [27]. In primate eyes, the short posterior ciliary arteries and branches of the ophthalmic artery form a circle around the optic nerve as they pass almost perpendicularly through the sclera to supply the choroid [28]. The short posterior ciliary arteries supply the posterior choroid and the long posterior ciliary arteries supply the anterior portion of the choroid.

The development of human choriocapillaris is unusual when compared with other capillary beds in the body [29]. Choriocapillaris develops before the formation of intermediate or large vessels, via hemovasculogenesis in which blood vessel and blood cells differentiate from a common precursor, the hemangioblast [24]. In humans, the choriocapillaris starts to differentiate simultaneously with the development of the RPE during the fourth and fifth week of gestation. At this stage of development, cells expressing CD31, CD34, CD39 and vascular endothelial growth factor receptor 2 (VEGFR-2) are detected where choriocapillaris will form [23]. Later, at around 11 and 12 weeks of gestation, the development of intermediate choroidal vessels in Sattler’s layer occurs by angiogenesis, facilitating the connection of choriocapillaris with the larger vessels of the Haller’s layer [24]. The choriocapillaris is fully mature, with flat, thin-walled fenestrated vessels at 22 weeks of gestation [30].

Alterations in choroidal structure or impaired blood flow result in degenerative changes and neovascularization, such as choroidal coloboma and AMD [31,32]. The thickness of the choroid can be used to assess abnormalities in choroidal vasculature, and it has been proposed as a biomarker for cardiovascular diseases [26]. There is an increasing interest in the development of quantitative methods to assess choroidal structural characteristics and their associations with ocular diseases (see Section 4).

Retinal blood vessels are organized in two planar layers that are restricted to the inner layers of the retina. The central retinal artery enters the eye through the centre of the optic nerve. The artery then branches in the inner retina to form three capillary layers. The retinal vessels provide blood to the inner two-thirds of the retina [33]. The retinal vessels develop into intraretinal capillaries that ramify at the inner and outer plexiform layers [34]. In mammals, retinal vessels, as opposed to the choriocapillaris, are not fenestrated and nourish the retina mainly by transcytosis of nutrients, since the presence of tight junctions between the endothelial cells restrict paracellular diffusion. Retinal pericytes directly contact the vascular endothelium. Pericytes are enclosed by the basement membrane and abundantly express smooth muscle actin that confers contractile properties [35].

Early in eye development, the oxygenation of the retinal tissue is provided by the vascular networks of the choroid and hyaloid. Hyaloid vessels progressively regress by apoptosis while the development of the retinal vasculature occurs on an astrocyte scaffold [36]. Failure of this regression is associated with incomplete retinal vascularization [37], suggesting an interplay of these two mechanisms. In humans, the formation and regression of the hyaloid vasculature and most of the retinal vasculature development occur in utero. In mice, retinal vasculature development is similar to humans but begins postnatally [38]. The most superficial retinal vascular layer is the first to be formed, starting from the optic nerve head and progressing toward the peripheral edge of the retina. When this superficial layer is almost formed, retinal vessels grow into the retina to form the deep retinal vascular layer at the base of the outer plexiform layer. Then, the intermediate layer forms between the superficial and deep layers, superficial to the inner plexiform layer, building a well-organized network to complete the three vascular layers [30]. Each of these three layers has a characteristic location and branching pattern and is considered an independent neurovascular unit.

Angiogenesis is the process by which proliferating endothelial cells form new vessel sprouts and extend the vascular network from pre-existing vessels. Vasculogenesis is defined as the de novo formation of blood vessels from isolated vascular endothelial precursor cells that coalesce into cords and then form a lumen. Historically, there has been controversy regarding the development of retinal vessels. Some authors support that retinal vasculature is formed by angiogenesis [34,36,39,40] while others consider that vasculogenesis is the process underlying the formation of retinal vasculature [41,42,43]. Moreover, some authors are proposing that retinal vasculature develops in two steps: the initial formation of the primary internal vascular network by vasculogenesis, followed by an angiogenic phase that founds the deep secondary network.

Non-endothelial cells, such as astrocytes and retinal ganglion cells, provide key molecular and structural cues for the developing retinal vasculature. Astrocytes, retinal ganglion cells and cells in the inner nuclear layer express vascular endothelial growth factor (VEGF) [44]. In particular, VEGF-A is secreted by astrocytes and retinal ganglion cells to promote new vessel formation and to alleviate tissue hypoxia [45]. Apparently, VEGF-A originated from astrocytes are not required for angiogenesis [45]. Nevertheless, astrocytes provide structural “paths” for endothelial cells to use as a template as the vascular network expands [46]. Recently, the VEGF receptor Flt-1 was demonstrated to regulate the extent of vascular growth and to promote the efficiency of endothelial tip cells and their filopodia in establishing new vessels in spatially defined locations along the astrocyte “template” [47]. VEGF isoforms have been shown to perform specific functions during the development of the vasculature [48,49]. For example, the VEGF_164_ isoform (in the mouse; VEGF_165_ in humans) leads to leukocyte adhesion in pathological, but not the physiological, neovascularization [50]. The complete inhibition of intracellular VEGF signaling results in substantial suppression of normal vascular development [50,51], suggesting that VEGF isoforms differently to VEGF_164_, and in combination, may be sufficient to promote normal physiological neovascularization. Despite the high expression of VEGF by retinal ganglion cells in the developing macula [52], the migration of endothelial cells and astrocytes in the developing macula is inhibited and the fovea never develops a retinal vasculature [53]. This feature is probably due to the expression of high levels of anti-angiogenic factors, such as the pigment epithelium-derived factor (PEDF) [54]. Axon guidance factors of the ephrin, semaphorin, slit and netrin families may also have a role in regulating vascular growth in the macula through repellent mechanisms [55].

## 3. The Role of Microglia in Retinal Vascular Development

In the retina, vascular patterning is essentially dependent on astrocytes in physiological conditions [56]. Astrocytes secrete VEGF in response to hypoxia, guiding the endothelial cells. Other cells also participate in the development of retinal vasculature. For example, modulating the HIF/VEGF system in horizontal or amacrine cells changes the density of the adjacent capillary plexus [57], demonstrating that these cells are also required for generating and maintaining the intraretinal vasculature.

Microglia are the resident innate immune cells of the central nervous system with crucial functions during development, and in normal and pathological conditions [58]. In the adult healthy, microglia keep a fairly low turnover rate without contributions from cells of the periphery, such as circulating monocytes or blood-derived macrophages [59]. These cells have important roles in health and disease and in development, including the modulation of retinal angiogenesis [60]. Although microglia and the vascular cells do not appear in the developing retina at the same time, the association of microglia with the developing retinal vasculature has been described for many years [61]. This association is supported by experimental data showing that pharmacological depletion of resident microglia from the developing retina, decreases retinal vascular area and density, as the effect that is amended by the reintroduction of microglial cells in the vitreous [60].

**Figure 1:**
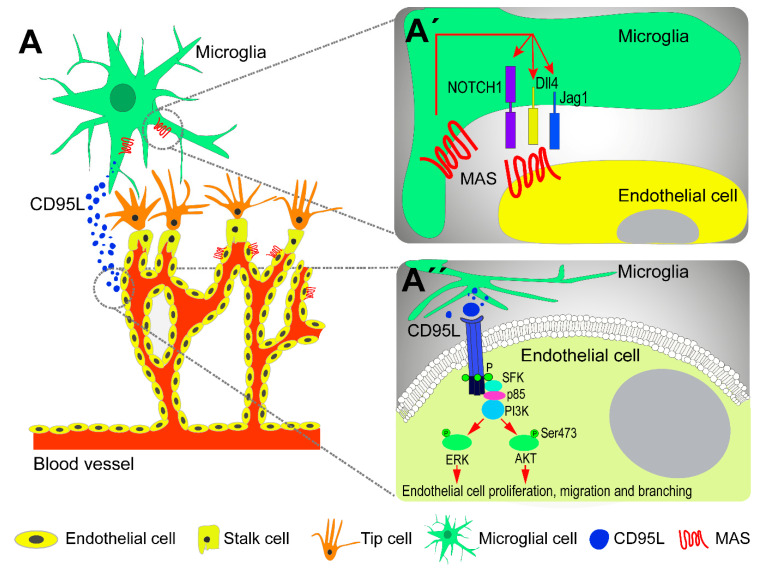
Contribution of microglial cells to vascular development in the retina. (**A**) The activation of the MAS receptor is involved in the recruitment of microglia and vascular growth in the developing retina by activating the Notch signaling pathway. (**A’**) Microglial cells secrete CD95L that binds to its receptor CD95 on endothelial cells. The phosphorylation of the death domain by Src-family kinase results in the activation of PI3K signaling pathways, leading to the activation of AKT or ERK (**A’’**).

Microglia, present at sites of endothelial tip cell anastomosis, stimulate vessel sprouting. However, physical contact between microglial and endothelial cells is not essential for the angiogenic stimulatory effects of microglia, suggesting that the effects of microglial cells on retinal vasculature are mediated via soluble factors secreted by these cells. Indeed, microglia secrete soluble factors that shape vascular growth and branching [62], such as CD95L that activates CD95 on vessels, mediating vascular growth through Src-family kinase and PI3K signaling [63] (Figure 1). Additionally, basigin-2, an extracellular matrix metalloproteinase inducer, was reported to facilitate microglia-endothelial communication through the secretion of insulin-like growth factor-1 (IGF-1) via the PI3K/AKT signaling pathway [64]. Recently, MAS, the receptor of angiotensin-(1-7), was shown to play an important role in microglia recruitment and vascular growth in the developing retina [65]. The activation of MAS causes the upregulation of Notch1, Delta-like ligand 4 and Jagged1 expression, members of the Notch signaling pathway (Figure 1). Indeed, this signaling pathway has been implicated in microglia localization and interaction with endothelial cells during sprouting angiogenesis and on the regulation of the fate of tip cells [66,67].

Interestingly, systemic inflammation in the neonatal period is known to impair vessel development by decreasing vessel extension, reducing capillary density and inducing localized overgrowth of abnormal retinal vessels [68]. In addition to the astrocytes that are localized in the lesion of abnormal vessels, activated inflammatory cells might cross the blood-retinal barrier and affect the normal vascular growth in the developing retina [68]. The infiltration of immune cells may be responsible for the increase in inflammation-related cytokines, such as tumor necrosis factor (TNF), interleukin (IL)-1β and IL-12a. TSP-1 is an anti-angiogenic and pro-apoptotic factor of retinal angiogenesis during development that antagonizes VEGF-mediated signaling [69]. TSP-1 was reported to be increased in systemic inflammation-induced retinopathy [68]. Moreover, microglia are also implicated in abnormal retinal vascular development during early postnatal inflammatory stress. These cells become reactive and are found either around branch points of sprouting vessels or at tip cells [70]. These alterations impact retinal neuronal function later in life, probably due to exacerbated microglial activity as reflected by the increase in *IL-1β*, *IL-6* and *TNF*, implicating microglia as the cellular player by which perinatal inflammation causes visual deficits [70].

## 4. Changes in Retinal and Choroidal Vascular Structure and Function in Age-Related Macular Degeneration (AMD)

Age-related macular degeneration is a leading cause of vision loss among the elderly population in developed countries [71]. The global prevalence of AMD is expected to increase from 196 million people in 2020 to 288 million in 2040, as a consequence of exponential ageing [72]. This disease affects the central region (macula) of the retina, as a result of photoreceptor/RPE/Bruch’s membrane/choriocapillaris complex abnormalities. When the central area of the macula, named the foveal avascular zone (the area containing the highest density of cones) is affected, the central field of vision of patients becomes compromised [73,74]. Age-related macular degeneration is a degenerative disease that progresses from early and intermediate AMD, which are mainly characterized by the accumulation of yellowish deposits called drusen located beneath the RPE and abnormalities of the RPE, respectively, to late-stage AMD defined by severe retinal and choroidal damage [75,76].

Age-related macular degeneration is a leading cause of vision loss among the elderly population in developed countries [71]. The global prevalence of AMD is expected to increase from 196 million people in 2020 to 288 million in 2040, as a consequence of exponential ageing [72]. This disease affects the central region (macula) of the retina, as a result of photoreceptor/RPE/Bruch’s membrane/choriocapillaris complex abnormalities. When the central area of the macula, named the foveal avascular zone (the area containing the highest density of cones) is affected, the central field of vision of patients becomes compromised [73,74]. Age-related macular degeneration is a degenerative disease that progresses from early and intermediate AMD, which are mainly characterized by the accumulation of yellowish deposits called drusen located beneath the RPE and abnormalities of the RPE, respectively, to late-stage AMD defined by severe retinal and choroidal damage [75,76].

Although drusen biogenesis is not fully understood, some authors have suggested that drusen result from the RPE or choriocapillaris damage. The specific mechanisms that connect RPE and choroidal endothelial cells pathology and drusen formation may include oxidative injury from light exposure or systemic factors, like compounds associated with smoking, lipofuscin accumulation, complement activation, Bruch’s membrane-induced dysfunction and ischemia [32,77,78,79,80,81,82,83,84]. Drusen are made up of a complex mixture of inflammatory mediators and lipids of retinal and choroidal origin [77,85,86,87,88,89] and their number and size may be indicative of risk for some future vision loss. Small drusen with well-demarcated borders (hard drusen) are usually neither age-related nor associated with an increased risk for the development of neovascularization [90,91], while larger drusen (measuring 63 μm or greater) lacking distinct borders (soft drusen) predict progression to its advanced forms of the disease [92].

Besides subretinal drusenoid deposits found in AMD, several histopathological studies reported the presence of yellowish lesions in the fundus, which can be viewed using blue light. Although these reticular pseudodrusen have some similarities in their composition compared to the subretinal deposits, such as the presence of vitronectin, complement proteins, apolipoprotein E and unesterified cholesterol, they lack immunoreactivity for protein markers of RPE, Müller glial and photoreceptor cells [93,94]. Interestingly, the presence of reticular pseudodrusen has been associated with late manifestations of AMD, including both geographic atrophy (nearly 20% of patients) and choroidal neovascularization (about 43% of patients) [95,96]. The geographic (dry) form of AMD is hallmarked by the presence of drusen and atrophy of the RPE. The exudative (wet) form is characterized by the growth of abnormal and fragile vessels from the choroid (known as choroidal neovascularization) under and into the macular portion of the retina. The leakage of blood and fluid from these newly formed vessels (choroidal neovascular membranes) contribute to the damage of the macula and cause central vision to become blurred and distorted. Although exudative (wet) AMD is less common (10 to 15% of affected individuals) than the dry form of the disease, it is associated with a faster sight decline compared to dry AMD, in which the rate of vision loss is usually very gradual.

With the help of OCTA, that allows the study of retinal and choroidal microvasculature and en face visualization of the blood flow at different anatomic retinal layers, without the need for the dye injection [97], it is becoming increasingly clear that AMD pathogenesis may extend beyond the outer retina. In fact, although intermediate AMD is hallmarked by the presence of at least one large druse (>125 μm) and abnormalities in RPE or both, it has been reported that the inner retinal vasculature is also affected in intermediate AMD [98]. Eyes of AMD patients present reduced vascular density in the superficial capillary plexus and decreased total vessel length and average vessel diameter in the deep capillary plexus, suggesting an association between density changes and decreased vessel number and caliber [98,99]. The complexity of the vasculature is also reduced in both capillary layers, which suggests loss or reduced flow of vessels at the intermediated AMD stage [99]. Besides alterations in retinal vessels, several other structural changes seem to be present in the inner retina in the early stages of AMD, such as loss of GCL, and inner plexiform layer and ganglion cell complex thickness [100,101,102,103,104,105,106,107]. In fact, a decrease in GCL thickness, in intermediate AMD, seems to be associated with changes in the vasculature supplying the inner retina [99], resulting in ischemia and cell loss. Ganglion cell complex thinning and photoreceptor cell damage (measured by the reflectivity of the en face inner segment/outer segment junction disruption) were found in studies investigating the relationship between ganglion cell complex thickness and photoreceptor alterations in eyes of patients with intermediate AMD [101]. Moreover, decreased thickness of inner retinal layers and peripapillary retinal nerve fiber layer correlates with AMD [100]. Although these reports suggest correlations between outer and inner retinal changes, it is difficult to ascertain the exact relationship between structural and vascular components of the inner retina in AMD.

With distinct approaches, several studies demonstrated the association between choroidal vascular changes with ageing and early AMD. Fluorescein angiography showed a prolonged choroidal filling phase in patients with early AMD [108], which is in agreement with the observations of reduced choroidal perfusion caused by a change in diffusional characteristics of the Bruch’s membrane [109,110]. Choroidal thickness is negatively correlated with age [111], and fluorescein angiograms show reduced blood volume and abnormal blood flow in eyes with nonexudative AMD [90,112]. A combination of choriocapillaris luminal narrowing, loss of cellularity and thinning of the choroid has been proposed as a potential cause of reduced blood flow [113]. Moreover, choriocapillaris dropout has been well documented in AMD patients and is usually associated with morphological features of eyes with AMD, including the accumulation of drusen, presence of reticular pseudodrusen and RPE atrophy [32,114,115,116].

In the case of neovascular AMD, the disease is hallmarked by choroidal neovascularization and associated manifestations such as pigment epithelial detachment, retinal pigment epithelial tears, disciform scarring and intraretinal hemorrhages [117]. The choroidal neovessels breach the Bruch’s membrane and invade sub-RPE and subretinal spaces. Clinical assessment of neovascular AMD is based on visual acuity testing, Amsler grid testing and slit-lamp examination, and in certain cases, to ascertain whether the disease is active, spectral-domain (SD)-OCT and fundus fluorescein angiography (FFA) are used. In fact, with fundus fluorescein angiography (FFA), the gold standard for the diagnosis of neovascular AMD, in combination with SD-OCT, that aids in both the diagnosis and follow-up of the disease, choroidal neovascularization (CNV) can be classified into three types: type 1 CNV, which involves the sub-RPE space and refers to vessels beneath the RPE (corresponds to angiographically occult CNV); type 2 CNV, which also involves the sub-RPE space and refers to neovessels growing from the choroid to the subretinal space between the neurosensory retina and the RPE (corresponds to angiographically classic CNV); and, type 3 CNV, which appears as intraretinal anastomosis originating in the deep capillary plexus of the retina [117].

## 5. The Contribution of Microglia to Retinal and Choroidal Neovascularization in AMD

In normal retinas, the continuous surveillance for the detection of noxious stimuli is performed by microglia, which are mostly confined to the plexiform layers where they exhibit complex ramified processes sensing the local retinal microenvironment [118,119]. These cells play an important role in retinal homeostasis, contributing to neuroprotection against transient pathophysiological assaults. Microglia express a variety of markers, such as CD45, MHC-I, MHC-II and macrophage antigens, including Iba-1, which suggests that microglia are a heterogeneous population of cells [118,120]. Inflammatory responses during retinal pathophysiology are coordinated by microglial cells [121].

Several mechanisms are known to be involved in endothelial dysfunction in the retina and choroidal neovascularization in AMD, such as oxidative stress and chronic inflammation [122]. Nitric oxide is produced by three nitric oxide synthase isoforms (endothelial, neuronal and inducible) that are expressed to variable degrees in the retina [123]. In the retina, nitric oxide is required for normal visual function. Although nitric oxide is itself a radical, its reactivity is low compared to the oxidative products, for example, dinitrogen trioxide (N_2_O_3_) and peroxynitrite (ONOO^−^), can be generated in the presence of concomitant oxidative stress [124]. This can lead to nitrosative stress following the reaction of these reactive products with molecules, such as proteins, lipids and DNA [125,126]. Oxidative and nitrosative stress, as a result of an imbalance between the production of reactive oxygen and nitrogen species, and antioxidant defense system plays a key role in the onset and progression of AMD [127,128,129,130,131].

The retina is particularly prone to oxidative stress since it is the most oxygen-consuming tissue in the body [132] and most of the oxygen consumption occurs in photoreceptor and RPE cells. RPE cells are responsible for phagocytosing and shedding photoreceptor outer segments. With age, the phagocytic capacity of RPE cells, that is essential for the renewal of photoreceptors (rods and cones), is compromised, and incompletely degraded material is deposited in the form of lipofuscin in Bruch’s membrane, contributing to drusen formation and Bruch’s membrane thickening [133,134,135,136,137]. Impaired clearance mechanisms of RPE, as a result of the excessive amount of reactive oxygen species and oxidative damage to DNA, proteins and lipids, can contribute to increased lipofuscin (the main constituent of drusen made of free-radical-damaged protein and fat) accumulation.

**Figure 2:**
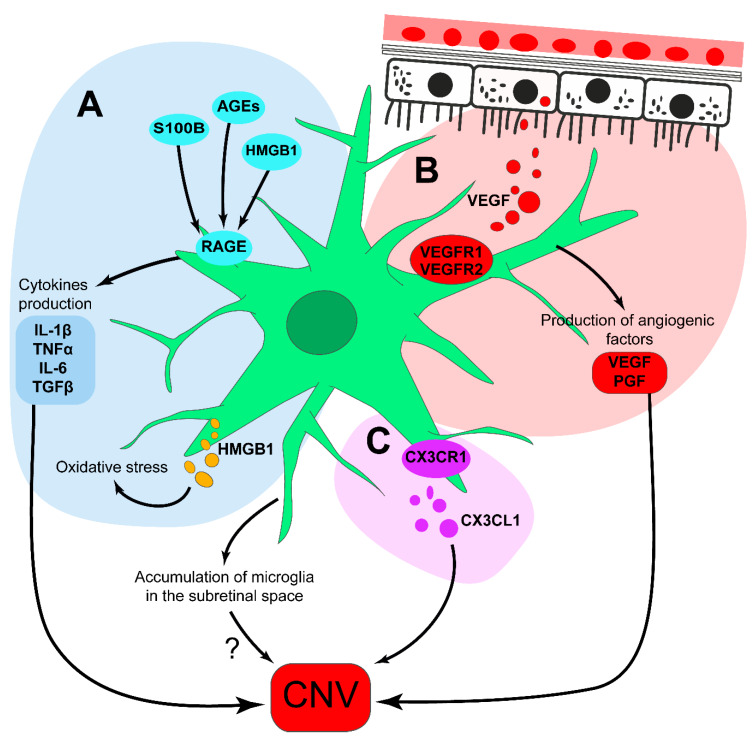
The role of microglial cells in choroidal neovascularization (CNV). Microglial AGE receptor (RAGE) activation by RAGE ligands (AGEs, S100B and HMGB1) leads to the release of inflammatory cytokines (IL-1β, TNFα, IL-6 and TGFβ) and production of ROS, ultimately leading to CNV (**A**); VEGF secreted by RPE can activate microglial VEGFR1 and VEGFR2, which may play a role in the early and late stages of CNV (**B**). Moreover, proangiogenic cytokines and growth factors like VEGF and PGF produced by microglia contribute to CNV (**B**); interaction of CX3CR1 with its ligand CX3CL1 may play a role in regulating microglia accumulation in the subretinal space, retinal degeneration and ultimately to CNV (**C**).

In an environment of oxidant stress, as it occurs in aged RPE, production and accumulation of advanced glycation end product (AGEs) are enhanced, as well as, activation of AGEs receptors (RAGE) [138], which are found in endothelial cells, pericytes, microglia, monocytes and macrophages, among other cells [138]. Experimental studies have shown that exposure of RPE cells to RAGE ligands, AGEs or S100B, can lead to retinal tissue damage, through RPE-mediated VEGF expression, leading to pathologic angiogenesis [139,140]. Although the receptor for AGEs is not usually expressed in high levels in the retina, it was found to be highly accumulated in RPE cells, photoreceptors and choriocapillaris in advanced AMD [141,142]. RAGE is recognized as a pattern-recognition receptor, and in addition to binding AGEs, can bind other proteins, such as high mobility group protein B1 (HMGB1), which can be released by necrotic cells passively, and by active secretion from macrophages, natural killer cells, and dendritic cells. Interaction between RAGE and its ligands results in a wide range of effects on several cellular pathways that are important in oxidative stress and inflammation [143,144,145,146,147]. RAGE activation, as a result of its interaction with S100B, was shown to contribute to CNV through regulating angiogenic activity, immune cells (microglia/macrophages) activation and infiltration to the damaged site, and upregulation of pro-inflammatory cytokines [148] (Figure 2).

It is known that drusen components, such as Aβ peptide 1–40 may be responsible for the increased expression of inflammatory molecules and inflammasome components in the retina and RPE in AMD. Furthermore, several cytokines (including TNF and IL-1β, IL-6 and transforming growth factor-beta (TGF-β)) have an important role in CNV [149]. A recent report has shown that pro-angiogenic cytokines and growth factors like VEGF and placental growth factor (PGF), which are produced by microglia (and macrophages), are present in high levels in ocular fluids of AMD patients [150]. Blockage of these molecules using antibodies reduces neovascularization and leakage, in a laser-induced CNV mouse model [150]. Furthermore, IL-1β levels are strongly reduced after PGF and VEGF-A co-inhibition. Abolishment of IL-1β signaling through Il1r1 deficiency leads to a reduction in the number of CNV lesions both in a rat model of laser-induced CNV [151] and in a mouse model of AMD [152]. Altogether, these data suggest that modulation of the pro-inflammatory state governed by microglia by decreasing the expression of PGF can have an impact on choroidal neovessels formation. Moreover, increased levels of IL-6 have been found in a laser-induced CNV mouse model [153]. IL-6 receptor blockade significantly reduced the expression of MCP-1/CCL2, VEGF and inhibited macrophage infiltration into CNV areas [153]. TGF-β is mainly produced by RPE cells and pericytes [154] and has been implicated in the regulation of endothelial cell proliferation (activated angiogenesis), in macrophage infiltration, as well as in extracellular matrix (ECM) proteolytic remodeling (vascular remodeling) [155]. Increased levels of TGF-β are associated with retinal angiogenesis, through regulation of pro-angiogenic factors [156,157]. TGFβR2-deficient retinal microglia induce abnormal responses to laser-induced injury enhancing CNV, pointing out that the absence of TGF-β signaling in retinal microglia can contribute to neurodegeneration and neovascularization [22].

There is substantial evidence suggesting that changes in microglial cells are not merely associated with secondary phenomena in AMD. Besides the contribution of RPE dysfunction to the formation of the subretinal drusenoid deposits, an alternative or concurrent mechanism for drusen genesis has been proposed; an impaired recruitment of macrophages through a CC chemokine ligand 2 (CCL2) and CC chemokine receptor 2-dependent (CCR2-dependent) pathway from the choroidal circulation may hamper the clearance of age-related accumulation of debris in Bruch’s membrane [158].

Both CCL2- and CCR2-dependent macrophage recruitment plays a crucial role in the development of experimental CNV, and ocular-infiltrating macrophages present a direct angiogenic ability [159]. Based on the fact that microglial cells are not the predominant sources of CCL2 in the retina, other cells such as Müller glial cells promote the extravasation of monocytes through the retinal vasculature, and immune cell recruitment may contribute to dysregulation in retinal para-inflammation and AMD [159]. Nevertheless, microglial cells express the C-X-C-motif chemokine receptor 3 (CX3CR1; receptor for CX3C chemokine ligand 1 (CXC3CL1)), another chemokine receptor that regulates the responses of microglia during inflammation (Figure 2). It has been demonstrated that all retinal microglial cells express CX3CR1, and these cells accumulate subretinally in affected areas of the macula in AMD, suggesting the infiltration of the subretinal space by microglia cells [160]. However, in CX3CR1-deficient mice, accumulation of microglial cells is also observed in the subretinal space at sites of retinal degeneration and is associated with an exacerbation of CNV [161]. The recruitment of activated microglia to the milieu of drusen and atrophic lesions are thought to contribute to drusen formation, retinal degeneration and CNV [77,79,161,162,163]. Consistently, increased oxidative and nitrosative stress is associated with increased numbers of Iba1^+^ macrophages/microglia in the retina and choroid in AMD eye sections. These data are in agreement with observations that macrophages and microglia recruitment in the macula are strongly associated with both early and advanced AMD [164].

In AMD the complement system activation is compromised at the level of retinal microglia/macrophages, thus contributing to the onset and progression of AMD. Deposition of complement, including C3, in affected areas of RPE/Bruch’s membrane, is associated with the expansion of atrophic lesions. Interestingly, intravitreal injection of small interfering RNA (siRNA) can suppress the local production of C3 by macrophages, increasing retinal complement activation and degeneration [163]. Complement component 3 (C3d), which plays a key role in enhancing B cell-specific immune responses [165], has also been recently described in the subretinal space of aged CXCR5 knockout mice (CXCR5^−/−^) [166]. Aged CXCR5^−/−^ mice present retinal degeneration, with photoreceptor cell death, upregulation of TNF and breakdown of the outer blood-retinal barrier. Moreover, these animals exhibit drusen-like deposits and the presence of Aβ and Cryab, two abundant proteins that are present in Bruch’s membrane and choroidal tissues of AMD patients [167,168]. Aβ and Cryab induce activation of the alternative complement cascade and are a target for microglia adaptive immune responses [167,169].

Disturbance of the innate immune system in AMD is associated with dysregulated complement and inflammasome activation and reactive microglia [170]. Increased levels of complement fragments C3a and Ba, and cytokines (EGF, IL-1a2, IL-6, IL-8, ICAM1, MCP-1, among others) were found in aqueous humor samples of patients with exudative AMD [171,172]. Moreover, bioactive fragments of complement (C3a and C5a) present in drusen of AMD patients can induce VEGF expression, increasing susceptibility to CNV formation [173].

Studies carried out on mice lacking the hypoxia response element (HRE) in the VEGF promoter (Vegf^δ/δ^ mice) showed that CNV is almost totally prevented [174]. Although VEGF has a positive impact in wet AMD development, its receptors, VEGFR1 and VEGFR2, seem to play differential roles in regulating the recruitment and accumulation of retinal microglia/macrophage in the subretinal space. VEGFR1 plays an important role in the early stage of CNV, whereas both receptors play pivotal roles at the later stages, suggesting that the angiogenic response involves the two receptors. These data are associated with the regulation of two sub-populations: VEGFR1&2^+^CD45^+^CD11b^+^, which represent circulating cells responding to the early stage of an experimental model of laser-induced CNV, and VEGFR1&2^−^Iba1^−^, which represent the microglia in the retina. However, it remains to be clarified whether the activation of the former is needed to recruit the latter to injured sites [174].

## 6. Modulation of Microglial Cells as a Potential Treatment for Neovascular AMD

In this review, we summarized the pieces of evidence supporting the contribution of microglial cells and microglial-driven inflammation to neovascularization. Since inflammation and neovascularization are closely related, several research teams tested if modulation of microglia activity might prevent retinal neovascularization.

Ablation of microglial cells via PLX5622 administration, an inhibitor of the colony-stimulating factor-1 receptor, results in a faster decrease of the CNV lesion size [175], suggesting a direct contribution of microglia to the maintenance of the CNV lesion. A few other studies suggested that inhibition of microglia-driven inflammation might be beneficial for the treatment of neovascular AMD, these included the knockdown of tumor necrosis factor receptor-associated factor 6 (TRAF6), the inhibition of sialic acid-binding immunoglobulin-like lectin-11 (SIGLEC11) receptor and β2–adrenergic receptor. TRAF6 facilitates the inflammatory response in microglia and macrophages and promotes tumor angiogenesis via upregulating the expression of HIF-1a and VEGF [176]. Intravitreal administration of TFAF6 siRNA inhibits the activation of microglia and macrophages, the formation of CNV and the expression of HIF-1a and VEGF [177]. Sialic acid polymers prevent reactive oxygen species production by human mononuclear phagocytes via the SIGLEC11 receptor. Intravitreal injection of a low-dose of low molecular weight polysialic acid with an average degree of polymerization 20 (polySia avDP20) in humanized transgenic mice expressing SIGLEC11 on mononuclear phagocytes reduced their reactivity and vascular leakage induced by laser coagulation [178]. β2–adrenergic receptor signaling increases Vegf and IL-6 RNA expression in mouse retinal microglia. Intravitreal injection of an β2–adrenergic receptor antagonist reduces CNV by 35% and decreases IL-6 protein levels by approximately 50%, these effects being partially due to the blocking of this pathway on microglial cells [179]. Moreover, systemic delivery of IFN-β attenuates microgliosis and macrophage responses in the early stage of the disease and reduces CNV size in the late phase [180]. Intravitreal administration of IL-4 attenuates laser-induced CNV due to specific IL-4 conditioning of microglia/macrophages. IL-4 induces the expression of sFlt-1 by resident CD11b-positive retinal microglial cells and infiltrating myeloid cells [181].

While it is still difficult to be certain about the best strategy to modulate microglial cells and to prevent neovascularization, the treatment of neovascular AMD is currently performed by routine intraocular injections of anti-VEGF agents, and to some extent by photodynamic therapy and thermal laser [71]. The wide introduction of anti-VEGF therapy has led to an improvement in the prognosis of patients affected by neovascular AMD, resulting in more favorable outcomes for a previously blinding disease. Although all the previous benefits described, anti-VEGF therapy has its caveats, such as the probability of ocular infection, increased ocular pressure, cataract, vitreous hemorrhaging and retinal detachment [71]. Therefore, to overcome some of these limitations and side-effects, several drugs are being tested as adjuvants to anti-VEGF regiments, including a 2-mer pegylated DNA aptamer that selectively binds to PDGF-BB and PDGF-AB homodimers and heterodimers [182] (NCT01940900), and steroids such as triamcinolone acetonide [183] (NCT01249937) and dexamethasone [184] (NCT01243086). In contrast to anti-VEGF drugs that require regular eye administrations, gene therapy medicines provide sustained delivery of the therapeutic protein or peptide with a single-dose administration. It also has the advantage of being delivered to the target cell avoiding potential side effects. Several gene therapy trials for neovascular AMD where the transduced cell overexpresses an angiostatic protein to arrest CNV are ongoing. Amongst these are gene therapy delivering sFLT01, a fusion protein composed of VEGF/PlGF (placental growth factor) binding domain of human VEGFR1/Flt-1 (hVEGFR1) fused to the Fc portion of human IgG1 through a polyglycine linker [185] (NCT01024998), the lentiviral vector expressing endostatin and angiostatin [186] (NCT01301443), the adeno-associated viral vector expressing sCD59 [187] (NCT03585556) and the adenovirus vector coding PEDF [188] (NCT00109499). Short interference RNAs (siRNA) have been also exploited as a treatment for neovascular AMD. Clinical trials using single intravitreal injections of a chemically modified siRNA targeting VEGFR-1 (Sirna-027, also known as AGN211745), were performed. However, the last clinical study terminated early due to company decision (non-safety related) (NCT00363714; NCT00395057) [189].

## 7. Conclusions

In this review, we summarized the substantial research effort to identify and study new molecular pathways responsible for the vascular alterations observed in AMD. Although our knowledge is limited, it is becoming clear that the resident immune cells of the retina, the microglial cells, do mediate processes related to neovascularization in AMD. Evidence suggests that the contribution of microglial cells might be either indirectly through the secretion of pro-inflammatory cytokines or directly via the release of angiogenic factors. A better understanding of the role of microglia in neovascularization will allow us to develop new therapeutic modalities based on the modulation of microglia physiology and reactivity. It is then crucial to clarify how, when and for how long we should modulate microglial activity, and only new studies in innovative disease models will allow us to answer these questions.

The development of groundbreaking innovations in diagnostic technologies, such as OCTA, that allows unprecedented high-resolution visualization of alterations in the choroidal and retinal vasculature, in combination with new therapeutic approaches, some of them based on the modulation of microglial cells, open new perspectives for early disease detection methods and treatment options thus allowing the prevention of macular degeneration and consequent vision loss.
